# Evaluation of an automatic gas chromatographic system for the identification of bacterial infective agents

**DOI:** 10.1155/S1463924689000398

**Published:** 1989

**Authors:** C. Arcelloni, A. Griffini, R. Paroni, P. A. Bonini

**Affiliations:** 1 Istituto Scientifico H. S. Raffaele Via Olgettina 60 Milan 20132 Italy; 2 Istituto di Biochimica e di Chimica Facoltà di Agraria Università di Milano Milan Italy

## Abstract

The potential clinical application of gas chromatography to microbial identifcation was evaluated. A completely automated system, the MIS (Microbial Identification System; Hewlett- Packard) can analyse and identify pure strains by comparison of their cellular fatty acids patterns (C_9_-C_20_) with the reference parameters stored in a library. Three hundred and sixty-seven strains were tested, comparing the gas chromatographic results with those obtained by the traditional microbiological methods in the bacteriology laboratory of our Institute. A standardized extractive procedure was followed to obtain the fatty acid methyl esters (FAMEs), but some modifications to the recommended procedure were introduced in the bacterial growth procedures: colonies harvested not only from the recommended growth media but also from selective media routinely used in the bacteriology laboratory were successfully examined. These modifications did not influence the results but improved the ease for the user; good agreement with the comparison method was observed as far as identifications of genus and species are concerned for 238 cases. The major advantages of this computerized system are a reduction in the time required to obtain the final results, the elimination of human errors by using the autosampler and a better inter-laboratory comparability of results owing to a higher degree of objectivity. On the other hand, the limited throughput of MIS (only 40 samples in 24 h) prevents its use in a large routine laboratory; this technology is appropriate in emergency cases, in taxonomic studies and as a confirmatory method.


Journal of Automatic Chemistry Vol. 11, No. 5 (September–October 1989), pp. 191–200

### Evaluation of an automatic gas chromatographic system for the identification of bacterial infective agents

**C. Arcelloni, A. Griffini,* R. Paroni and P. A. Bonini**
*Istituto Scientifico H. S. Raffaele, Via Olgettina 60, 20132 Milan, Italy*

*The potential clinical application of gas chromatography to microbial identification was evaluated. A completely automated system, the MIS (Microbial Identification System; Hewlett-Packard) can analyse and identify pure strains by comparison of their cellular fatty acids patterns ($C_9$–$C_{20}$) with the reference parameters stored in a library. Three hundred and sixty-seven strains were tested, comparing the gas chromatographic results with those obtained by the traditional microbiological methods in the bacteriology laboratory of our Institute. A standardized extractive procedure was followed to obtain the fatty acid methyl esters (FAMEs), but some modifications to the recommended procedure were introduced in the bacterial growth procedures: colonies harvested not only from the recommended growth media but also from selective media routinely used in the bacteriology laboratory were successfully examined. These modifications did not influence the results but improved the ease for the user; good agreement with the comparison method was observed as far as identifications of genus and species are concerned for 238 cases. The major advantages of this computerized system are a reduction in the time required to obtain the final results, the elimination of human errors by using the autosampler and a better inter-laboratory comparability of results owing to a higher degree of objectivity. On the other hand, the limited throughput of MIS (only 40 samples in 24 h) prevents its use in a large routine laboratory; this technology is appropriate in emergency cases, in taxonomic studies and as a confirmatory method.*

### Introduction

Traditional techniques for the identification and classification of microbial infective agents are based on morphological, inmmuno-biochemical and physiological characteristics. Sometimes these parameters are insufficient to classify some strains and, according to Kreig [1], some of the routine methods so far used, especially for anaerobic cultures, are expensive and time consuming. Further, biochemical methods have a very variable discriminatory power, giving poorly comparable results. Moss [2] showed that the gas chromatographic analysis of metabolic products or of bacterial cell components offer a good tool in clinical microbiological laboratories for identifying the infective agents and for studying the taxonomic classification of bacteria. Goodfellow and Minnikin [3] and Brondz and Olsen [4] recently introduced new criteria for classifying the microorganisms on the basis of the proteic, lipidic and saccharidic composition of the bacterial cell.

The lipidic components of the bacterial envelope were particularly studied as specific markers for many strains: in Gram-positive bacteria, the cell lipids are concentrated in the plasma membrane whereas in Gram-negative bacteria lipoproteins and polar and non-polar lipids are located in the plasma or in the outer membrane. The chemotaxonomic classification of Gram-positive bacteria could be based only on the cellular fatty acid pattern because the metabolic products of these bacteria (ketones, alcohols and amines) are not specific enough [3]. However, Brondz and Olsen [4] and Drucker [5] reported that short-chain (1–7 carbon atoms) and non-hydroxylated fatty acids are specific components of the structure of anaerobic bacteria.

According to Asselineau and Asselineau [6], the introduction of fused-silica capillary columns with polar and non-polar stationary phases in gas chromatographic analysis has facilitated the identification of a large number of fatty acids and improved the resolution of this method for microbial identification.

The MIS (Microbial Identification System; Hewlett-Packard, Avondale, PA, USA) is a computerized and completely automated gas chromatographic apparatus for the identification of aerobic and anaerobic bacteria based on their cellular fatty acids composition ($C_9$–$C_{20}$). A pattern recognition program compares the fatty acids of an unknown sample with those of the reference bacteria stored in a computer library. The unknown strain is identified only if its fatty acids pattern has characteristics close to some of the patterns present in the library.

So far, the library contains the fatty acid patterns of many Gram-positive cocci, Gram-positive rods, Gram-negative cocci and Gram-negative fermenters and non-fermenters; it is expected to be updated for other anaerobic bacteria, yeasts, moulds and other fungi and mycobacteria.

In this paper, the potential application of the gas chromatographic MIS in clinical laboratories as a support for and/or alternative tool to traditional microbiological analyses is examined.

### Abbreviations

BA = blood agar; BHI = brain heart infusion agar; ECL = equivalent chain length; FAME = fatty acid methyl ester; FID = flame-ionization detector; GC = gas

* Istituto di Biochimica e di Chimica, Facoltà di Agraria, Università di Milano, Milan, Italy.

0142–0453/89 $3.00 © 1989 Taylor & Francis Ltd.

chromatography; HPLC = high-performance liquid chromatography; MCK = MacConkey agar; MIS = Microbial Identification System; MH = Müller–Hinton; MSA = mannitol salt agar; PEA = phenyl ethyl agar; RT = retention time; SI = similarity index; KV = Schädler-KV agar; TSB = trypticase soy broth; TSBA = trypticase soy broth agar.

### Materials and methods

*Description of the microbial identification system*

The MIS consists of the following: HP 5890A gas chromatograph (Hewlett-Packard), with methylphenyl-silicone fused-silica capillary column (25 m × 0·2 mm i.d.); flame ionization detector (FID); HP 3392A integrator; HP 9816S computer equipped with a 9133H disk drive; HP 2225A printer; and HP 7673A automated injector with sampler controller and sample tray for 100 vials.

*Operating conditions*

Ultra-high purity hydrogen (SIO-ALPHAGAZ) was utilized as the carrier gas. The column head pressure was 10 lb $in^{-2}$, the injector temperature was 250 °C and the detector temperature was 300 °C. Other operating parameters were: FID air, 400 ml $min^{-1}$; FID $H_2$, 30 ml $min^{-1}$; FID $N_2$, 30 ml $min^{-1}$; trap purge, 40 ml $min^{-1}$; septum purge, 5 ml min; splitting ratio, 100 : 1; and splitter, 50 ml $min^{-1}$.

At an initial oven temperature of 170 °C, a temperature program of 50 °C $min^{-1}$ was activated at injection and continued to a final temperature of 270 °C, which was held isothermal for 2 min. The time required for each run was 22 min and the re-equilibration of the column required 3 min.

*Extraction procedure*

All the reagents were of HPLC grade. The cellular fatty acids were extracted and derivatized following a standardized procedure. Reagent 1 was 45 g of sodium hydroxide (Merck, Darmstadt, FRG), 150 ml of methanol (Fluka, Buchs, Switzerland) and 150 ml of doubly distilled water. Reagent 2 was 325 ml of 6 M hydrochloric acid (Carlo Erba, Milan, Italy) and 275 ml of methanol. Reagent 3 was 200 ml of hexane (Merck) and 200 ml of diethyl ether stabilized with 2% ethanol (Merck). Reagent 4 was 10·8 g of sodium hydroxide in 900 ml of doubly distilled water.

Bacterial colonies were harvested with a 4-mm inoculating loop and coated at the bottom of the glass tubes (Pyrex, 14 × 100 mm) provided with Teflon-lined screw-caps. The amount of bacteria harvested with a double collection was sufficient for processing. A 1-ml volume of reagent 1 was pipetted into each tube, mixed for 5–10 s, heated at 100 °C in a block heater (Supelco, Bellefonte, PA, USA) for 5 min, mixed again and kept at 100 °C for 25 min.

The methylation of fatty acids was achieved by adding 2 ml of reagent 2 to the cooled uncapped tubes, which were then mixed for 5–10 s and heated at 80 °C for 10 min. The fatty acid methyl esters (FAMEs) were extracted by adding 1·25 ml of reagent 3 and shaking gently for 10 min on a laboratory rotator. The lower aqueous phase was removed with a Pasteur pipette and discarded. The upper phase was washed with 3 ml of reagent 4 and shaken gently for 10 min. Two thirds of each organic extract were transferred with a Pasteur pipette to the autosampler vials (Teflon caps) for the gas chromatographic analysis.

*Cultures*

In the bacteriology laboratory of our Institute, different aliquots of the same biological specimen were streaked as usual on four different media: blood agar (a non-specific medium for a quantitative evaluation of the bacteria), mannitol salt agar (MSA) (specific for the growth of Staphylococci, MacConkey Agar (MCK) for the identification of Gram-negative bacteria and Sabouraud medium for growth of fungi. These plates were incubated at 37 °C for 24 h. To detect anaerobic bacteria, specimens were streaked both on Schädler's medium for quantitative evaluation, on Schädlar-KV agar (KV) for identification of Bacteriodes and on phenyl ethyl agar (PEA) for Gram-negative cocci growth. The plates were then incubated at 37 °C in an anaerobic atmosphere until a suitable growth was obtained (2–5 days). After the primary isolation, both the aerobic and anaerobic strains were further characterized with microscopic, biochemical and serological tests. For the biochemical analyses API (Ayerst) and Enterotube (Roche) strips were used. The pure strains were subsequently transplanted on Müller–Hinton (MH) medium for the antibodies sensitivity test, according to Bauer *et al.* [7]. For our gas chromatographic study, the aerobic colonies were harvested directly from the specific media or from Müller–Hinton medium. For some aerobic strains, we used, according to the MIS recommendation, trypticase soy broth agar (TSBA) as a secondary medium, onto which the previously isolated colonies were transferred. This medium consists of 30 g of trypticase soy broth (BBL, Becton Dickinson), 15 g of Bacto agar (Difco, Detroit, MI, USA) and 1 l of distilled water. The ingredients were combined, boiled until the agar melted, autoclaved for 15 min at 121 °C (15 lb $in^{-2}$) and cooled to 60 °C, then dispensed into sterile Petri dishes. The cultures were incubated for 24 h at 28 °C. All the anaerobic bacteria examined in this study were harvested directly from KV or from PEA, without a secondary isolation on the media suggested by Hewlett-Packard.

*Samples analysed*

We studied 367 strains isolated from routine specimens of the bacteriology laboratory of our Institute. Biological specimens were represented by blood, catheter points, pus from deep wounds, swabs (from pharynx, rectum, vagina) and urine. All the samples were analysed both by the gas chromatographic technique and by classical microbiological procedures used as a reference method and the results were compared.

*Calibration for MIS*

For quantitative calibration, the FAMEs mixture for a

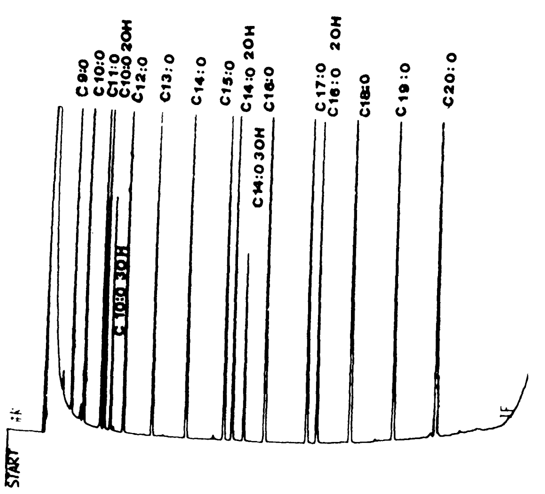

File: DATA:F0582340B

Bottle: 1

ID: 1

Name: Calibration Mix 1

Date of report: 13-SEP-87 13:33:21

Date of run: 23-AUG-87 11:51:02

Date edited: 23-AUG-87 17:40:13

**A**

| RT | Area | Ar/Ht | Respon | ECL | Name | Area % | Comment 1 | Comment 2 |
|---|---|---|---|---|---|---|---|---|
| 1.496 | 27263000 | 0.054 | . . . | 7.039 | SOLVENT PEAK . . . . . . | . . . | < min rt | |
| 2.443 | 66984 | 0.025 | 1.218 | 9.000 | 9:0 . . . . . . . . . | 5.08 | | |
| 2.926 | 142330 | 0.026 | 1.147 | 10.000 | 10:0 . . . . . . . . . | 10.16 | Peak match 0.0000 | |
| 3.541 | 942 | 0.026 | . . . | 10.915 | . . . . . . . . . . . . . | . . . | | |
| 3.598 | 74960 | 0.029 | 1.090 | 11.000 | 11:0 . . . . . . . . . | 5.09 | Peak match -0.0043 | |
| 3.734 | 30777 | 0.029 | 1.083 | 11.152 | 10:0 2OH . . . . . . | 2.07 | Peak match 0.0085 | |
| 3.973 | 15777 | 0.030 | 1.070 | 11.420 | 10:0 3OH . . . . . . | 1.05 | Peak match -0.0002 | |
| 4.491 | 157100 | 0.031 | 1.046 | 12.000 | 12:0 . . . . . . . . . | 10.23 | Peak match -0.0011 | |
| 5.616 | 79407 | 0.034 | 1.013 | 13.000 | 13:0 . . . . . . . . . | 5.00 | Peak match -0.0002 | |
| 6.512 | 1043 | 0.047 | . . . | 13.667 | . . . . . . . . . . . . . | . . . | | |
| 6.959 | 166320 | 0.037 | 0.987 | 14.000 | 14:0 . . . . . . . . . | 10.21 | Peak match 0.0003 | |
| 8.481 | 84469 | 0.040 | 0.968 | 15.000 | 15:0 . . . . . . . . . | 5.09 | Peak match -0.0041 | |
| 8.810 | 36251 | 0.042 | 0.965 | 15.200 | 14:0 2OH . . . . . . | 2.18 | Peak match 0.0093 | |
| 9.282 | 16903 | 0.043 | 0.960 | 15.486 | 14:0 3OH . . . . . . | 1.01 | Peak match -0.0019 | |
| 10.129 | 172580 | 0.043 | 0.954 | 16.000 | 16:0 . . . . . . . . . | 10.24 | Peak match -0.0007 | |
| 11.846 | 85705 | 0.045 | 0.943 | 17.000 | 17:0 . . . . . . . . . | 5.03 | Peak match -0.0029 | |
| 12.250 | 36753 | 0.047 | 0.941 | 17.231 | 16:0 2OH . . . . . . | 2.15 | Peak match 0.0043 | |
| 13.597 | 174890 | 0.048 | 0.935 | 18.000 | 18:0 . . . . . . . . . | 10.18 | Peak match -0.0014 | |
| 15.341 | 87936 | 0.047 | 0.929 | 19.000 | 19:0 . . . . . . . . . | 5.08 | Peak match 0.0001 | |
| 17.065 | 176510 | 0.049 | 0.925 | 20.000 | 20:0 . . . . . . . . . | 10.16 | | |

**B**

| Solvent Ar | Total Area | Named Area | % Named | Total Amnt | Nbr Ref | Ref ECL Shift | ECL Deviation |
|---|---|---|---|---|---|---|---|
| 27263000 | 1607637 | 1605452 | 99.88 | 1607050 | 0 | . . . . . . . . . | . . . . . . . . |

GOOD PEAK MATCHING: PEAK POSITION MATCHING ERROR (RMS) IS 0.0039.

**C**

*Figure 1. Gas chromatographic profile of a quantitative standard calibration mixture containing 17 fatty acid methyl esters (FAMEs). The upper part shows the GC profile and in the lower part the printed report is shown. Section A: Bottle, position of the sample in the sample-tray; ID, identification number of the various samples programmed in the Sample Table; Name, name of the sample programmed in the Sample Table. Section B: RT, peak retention time (in minutes); Area, peak absolute area; Ar/Ht, peak width at half-height; Respon, correction factor of the peak absolute areas obtained from the comparison with the peak area of an ideal standard mixture; ECL, equivalent chain length of the ideal standard calibration mixture (see text); Name, name of the fatty acid identified by the system; Area %, peak area as a percentage of the Named Area (see below); Comment 1, this section shows if the eluted peak has an RT in the range $C_9$–$C_{20}$ (peak match) or not (>RT or <RT); in the latter case the peak is not identified; the number reported on the right side of Peak match ± . . . indicates the shifting of the ECL obtained from the ideal one stored in the computer memory. Section C: Solvent Area, area of the hexane/ether peak; Total Area, amount of the peak areas eluted between $C_9$ and $C_{20}$; Named Area, size of the identified peak area, % Named, percentage of the total area; Total Amnt, product between the Area Named and the Respon factor; Nbr Ref, number of the peaks assumed as references; if present, these peaks are listed in Comment 2; obviously, this indication is used only for the unknown samples and not for standards.*

capillary column (Supelco) contains 12 straight-chain fatty acids (C9:0–C20:0) and 5 hydroxy acids (C10:0 2OH, C10:0 3OH, C14:0 2OH, C14:0 3OH, C16:0 3OH). The straight-chain fatty acids are used as references for the identification of the FAMEs in the bacterial samples, while the hydroxy acids are added to detect the column degradation, which is evident from tailed peaks. The injection of the quantitative calibration mixture was programmed in the Sequence Table of the MIS computer at the beginning of each batch of samples and after every 15 analyses.

Every 350 samples, three different qualitative calibration mixtures were injected. These mixtures are combined extracts of selected bacteria containing a different assortment of fatty acids (about 80 in each mixture). Some of these fatty acids are not available commercially and are specific for particular microorganisms only. The pattern recognition program could identify up to 150 different fatty acids.

*System programming*

After identification by the reference method, the samples chosen for the extraction, methylation and subsequent GC analysis were programmed by the user in a 'Sample Table,' according to the MIS computer software, and then analysed.

The qualitative and quantitative calibration mixtures injections were also programmed in the Sequence Table.

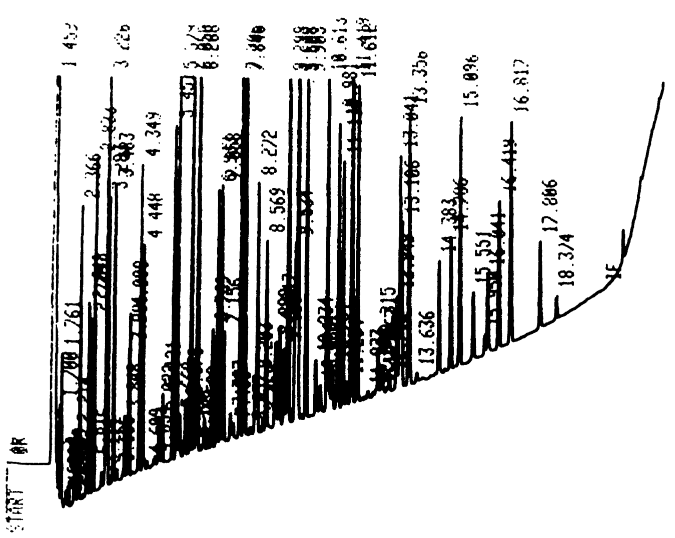

[illegible]: Microbial Identification System (Vers: 1.1) (Software s/n: 2611A100022 Computer s/n: 2510A21807)

*** CALIBRATION ***

File: DATA:F88308427

Bottle: 2

ID: 2

Name: QUALITATIVE CAL 2

Date of report: 08-MAR-88 12:24:10

Date of run: 08-MAR-88 12:24:10

**A**

| RT | Area | Ar/Ht | Respon | ECL | Name | Area % | Comment 1 | Comment 2 |
|---|---|---|---|---|---|---|---|---|
| 1.459 | 34679000 | 0.069 | ... | 7.059 | SOLVENT PEAK ........ | ... | < min rt | |
| 1.700 | 5464 | 0.027 | ... | 7.575 | ............. | ... | < min rt | |
| 1.761 | 10418 | 0.031 | ... | 7.705 | ............. | ... | < min rt | |
| 1.902 | 457 | 0.026 | ... | 8.007 | ............. | ... | < min rt | |
| 1.942 | 1069 | 0.027 | ... | 8.092 | ............. | ... | < min rt | |
| 1.985 | 670 | 0.023 | ... | 8.184 | ............. | ... | < min rt | |
| 2.039 | 1462 | 0.025 | ... | 8.300 | ............. | ... | < min rt | |
| 2.214 | 4546 | 0.027 | ... | 8.675 | ............. | ... | < min rt | |
| 2.366 | 19820 | 0.030 | 1.238 | 9.000 | 9:0 ........ | 1.40 | ECL deviates 0.000 | Reference 0.000 |
| 2.612 | 1793 | 0.023 | 1.182 | 9.527 | unknown 9.521 ... | 0.12 | ECL deviates 0.006 | |
| 2.648 | 11314 | 0.026 | 1.174 | 9.604 | 10:0 ISO ...... | 0.76 | ECL deviates -0.001 | |
| 2.729 | 9462 | 0.024 | ... | 9.777 | ............. | ... | | |
| 2.833 | 19192 | 0.026 | 1.139 | 10.000 | 10:0 ........ | 1.25 | ECL deviates -0.000 | Reference 0.000 |
| 3.114 | 1763 | 0.045 | ... | 10.433 | ............. | ... | | |
| 15.950 | 1948 | 0.043 | ... | 19.496 | ............. | ... | | |
| 16.041 | 9323 | 0.053 | 0.946 | 19.549 | 18:0 3OH ...... | 0.50 | ECL deviates -0.002 | |
| 16.419 | 15492 | 0.047 | 0.950 | 19.769 | 20:1 CIS 11 .... | 0.84 | ECL deviates -0.001 | |
| 16.817 | 24335 | 0.049 | 0.955 | 20.000 | 20:0 ........ | 1.32 | ECL deviates 0.000 | Reference -0.002 |
| 17.806 | 8548 | 0.044 | ... | 20.575 | ............. | ... | > max rt | |
| 18.374 | 2659 | 0.053 | ... | 20.905 | ............. | ... | > max rt | |

**B**

| Solvent Ar | Total Area | Named Area | % Named | Total Amnt | Nbr Ref | Ref ECL Shift | ECL Deviation |
|---|---|---|---|---|---|---|---|
| 34679000 | 2448110 | 1802935 | 73.65 | 1754962 | 12 | 0.001 | 0.003 |

**C**

*Figure 2. Gas chromatographic profile and analysis report of a qualitative calibration mixture. For the description of the analysis report, see legend of figure 1. In addition: Section B: in Comment 1 column, ECL Deviates, shifting of the obtained ECL from the ideal one; in Comment 2 column, Reference ± . . . , peaks used as reference to adjust the RT of the other peaks. The value (±) shows the direction of the adjustment given by the system. Section C: Ref ECL Shift, root mean square (RMS) of the adjustments made on the RT of the peaks listed in Comment 2; Deviation, standard deviation of the mean of the ECL shift listed in Comment 1.*

### Results

In figures 1 and 2 examples of chromatograms and analysis reports of the standard mixtures used for quantitative and qualitative calibration are shown. The GC profile is given during the run, while the analysis report is printed at the end. For an explanation of all the GC parameters reported, see the legends of the figures. The equivalent chain length (ECL) is a mathematical parameter calculated by the MIS software, which is very important for the interpretation of an unknown peak. This value corresponds to the number of carbon atoms in the fatty acid chain and allows the determination of the chemical structure of the unknown fatty acids eluting in the analysis. By convention, the $C_9$–$C_{20}$ straight-chain fatty acids were taken as reference points in the calculation of the ECLs of all the other fatty acids contained in the sample. The ECLs of the $C_9$–$C_{20}$ straight-chain acids

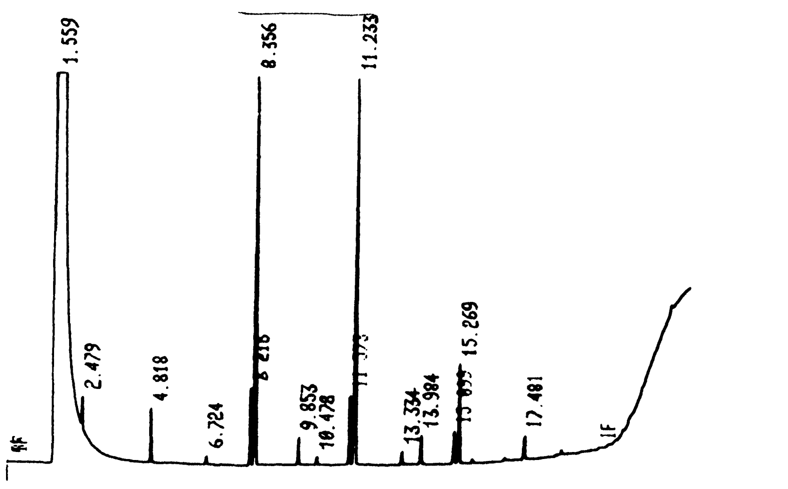

HP 5898A: MICROBIAL IDENTIFICATION SYSTEM (Rev: 2.1) (Sw s/n: 2711A10123) (Hw s/n: 2510A21 17-JUL-87 09:4

ID: 1154 STAPHILOCOCCUS M+D+C 989 Date of run: 17-JUL-87 14:50:02

Bottle: 54 SAMPLE [AEROBE]

| RT | Area | Ar/Ht | Respon | ECL | Name | % | Comment 1 | Comment 2 |
|---|---|---|---|---|---|---|---|---|
| 1.559 | 41319000 | 0.082 | . . . | 6.997 | SOLVENT PEAK . . . . . . . . | . . . | < min rt | |
| 4.818 | 4259 | 0.035 | 1.041 | 12.092 | 11:0 ISO 3OH . . . . | 2.97 | ECL deviates 0.002 | |
| 6.724 | 735 | 0.039 | 0.989 | 13.617 | 14:0 ISO . . . . . . | 0.49 | ECL deviates -0.001 | Reference 0.002 |
| 8.216 | 7120 | 0.041 | 0.965 | 14.621 | 15:0 ISO . . . . . . | 4.60 | ECL deviates 0.000 | Reference 0.001 |
| 8.356 | 72312 | 0.041 | 0.964 | 14.711 | 15:0 ANTEISO . . . . | 46.61 | ECL deviates 0.000 | Reference 0.001 |
| 9.853 | 2527 | 0.042 | 0.949 | 15.627 | 16:0 ISO . . . . . . | 1.60 | ECL deviates 0.001 | Reference 0.000 |
| 10.478 | 895 | 0.047 | 0.944 | 16.000 | 16:0 . . . . . . . . | 0.57 | ECL deviates -0.000 | Reference -0.001 |
| 11.573 | 7190 | 0.046 | 0.939 | 16.629 | 17:0 ISO . . . . . . | 4.52 | ECL deviates 0.000 | Reference -0.001 |
| 11.733 | 40103 | 0.046 | 0.939 | 16.721 | 17:0 ANTEISO . . . . | 25.18 | ECL deviates -0.001 | Reference -0.002 |
| 13.334 | 1421 | 0.048 | 0.936 | 17.632 | 18:0 ISO . . . . . . | 0.89 | ECL deviates -0.000 | Reference -0.002 |
| 13.984 | 3110 | 0.048 | 0.936 | 18.000 | 18:0 . . . . . . . . | 1.95 | ECL deviates -0.000 | Reference -0.002 |
| 15.099 | 3650 | 0.051 | 0.938 | 18.634 | 19:0 ISO . . . . . . | 2.29 | ECL deviates 0.001 | Reference -0.001 |
| 15.269 | 10763 | 0.048 | 0.938 | 18.730 | 19:0 ANTEISO . . . . | 6.75 | ECL deviates 0.001 | |
| 17.481 | 2504 | 0.049 | 0.948 | 20.000 | 20:0 . . . . . . . . | 1.59 | ECL deviates 0.000 | Reference -0.003 |

| Solvent Ar | Total Area | Named Area | % Named | Total Amnt | Nbr Ref | ECL Deviation | Ref ECL Shift |
|---|---|---|---|---|---|---|---|
| 41319000 | 156589 | 156589 | 100.00 | 149477 | 11 | 0.001 | 0.002 |

TSBA [Rev 2.0] Staphylococcus . . . . . . . . . . . . . . . . . . . 0.225

S. aureus . . . . . . . . . . . . . . . . . . . . 0.225 **S.I.**

S. a. aureus GC subgroup A . . . . . . . . . . 0.225

Comparison with TSBA [Rev 2.0]: Staphylococcus-aureus-aureus GC subgroup A Distance: 5.126

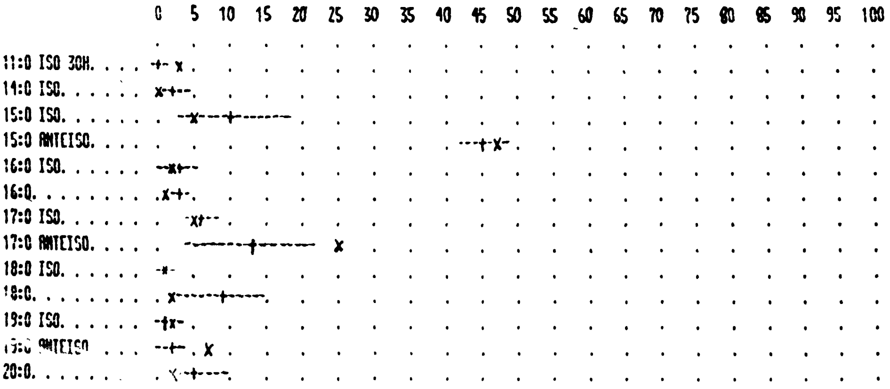

*Figure 3. Example of chromatograph, analysis report and comparison chart of pure strain of* Staphylococcus aureus *analysis. For the report see legends of figures 1 and 2. In addition: TSBA [Rev 2.0], identification name of the library in which the sample was searched;* Staphylococcus, *genus;* aureus, *species;* aureus *GC subgroup A, sub-species; 0·225 SI, the similarity index in this example is 0·225 (see text). The comparison chart gives the following information: on the left side all fatty acids found in both the unknown sample and in a library entry are listed in the elution order while a scale of % is printed across the top of the chart. Symbols:* x = *percentage of the fatty acid found in the sample; ---- = range of the percentage composition of the same acid in the reference bacteria; * appears when the percentages of the fatty acid in the sample and in the reference bacteria are identical.*

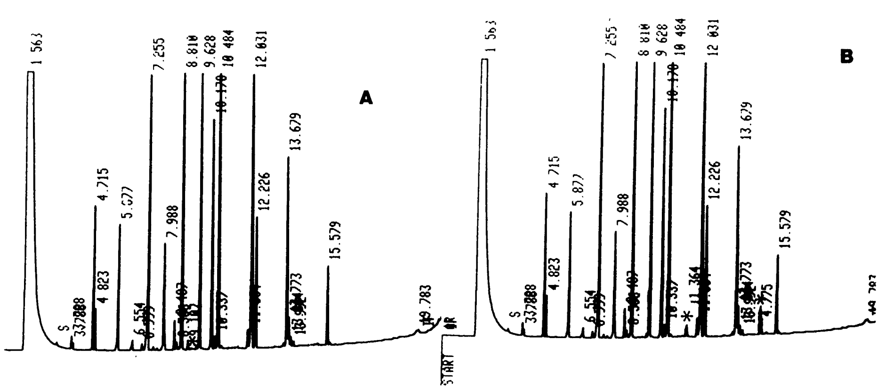

*Figure 4. In the upper part a chromatogram of one strain of* Escherichia coli *(A) and* Kluyvera cryocrescens *(B) obtained in our laboratory is reported. In the lower part, the 'ideal' (reported in the library) pattern of these microorganisms is reported.*

| | NAME | MEAN % | STD DEV | MIN % | MAX % |
|---|---|---|---|---|---|
| 9 | unknown 10.928 | 0.93 | 0.90 | 0.00 | 2.83 |
| 19 | 12:0 | 3.86 | 0.88 | 2.33 | 6.43 |
| 28 | 13:0 | 0.04 | 0.14 | 0.00 | 0.69 |
| 37 | 13:0 ISO 2OH | 0.03 | 0.09 | 0.00 | 0.31 |
| 39 | 14:0 | 6.69 | 0.91 | 5.31 | 8.90 |
| 46 | unknown 14.503 | 0.95 | 0.32 | 0.00 | 1.48 |
| 54 | 15:0 | 1.03 | 0.84 | 0.00 | 4.42 |
| 66 | 16:1 B * | 0.01 | 0.04 | 0.00 | 0.24 |
| 67 | 16:1 CIS 9 | 11.74 | 4.68 | 4.63 | 21.58 |
| 68 | 16:1 C | 0.02 | 0.07 | 0.00 | 0.30 |
| 69 | 16:0 | 29.14 | 2.48 | 24.72 | 36.66 |
| 83 | 17:1 B | 0.05 | 0.17 | 0.00 | 0.64 |
| 86 | 17:0 CYCLO | 12.59 | 3.32 | 5.17 | 18.93 |
| 88 | 17:0 | 0.35 | 0.47 | 0.00 | 1.65 |
| 101 | 18:0 | 0.13 | 0.32 | 0.00 | 1.67 |
| 110 | 19:0 CYCLO C11-12 | 3.94 | 2.88 | 0.00 | 12.23 |
| 124 | SUMMED FEATURE 3 | 6.83 | 1.17 | 4.60 | 10.96 |
| 128 | SUMMED FEATURE 7 | 21.67 | 4.90 | 12.53 | 36.70 |

| | NAME | MEAN % | STD DEV | MIN % | MAX % |
|---|---|---|---|---|---|
| 9 | unknown 10.928 | 1.44 | 0.34 | 0.98 | 1.96 |
| 19 | 12:0 | 4.63 | 0.77 | 3.52 | 6.26 |
| 28 | 13:0 | 0.25 | 0.40 | 0.00 | 1.09 |
| 37 | 13:0 ISO 2OH | 0.05 | 0.08 | 0.00 | 0.23 |
| 39 | 14:0 | 9.27 | 0.99 | 7.58 | 11.36 |
| 46 | unknown 14.503 | 0.72 | 0.43 | 0.00 | 1.19 |
| 54 | 15:0 | 1.79 | 1.96 | 0.00 | 5.57 |
| 67 | 16:1 CIS 9 | 14.36 | 4.97 | 6.60 | 21.70 |
| 68 | 16:1 C | 0.01 | 0.05 | 0.00 | 0.17 |
| 69 | 16:0 | 30.57 | 4.16 | 25.22 | 37.05 |
| 76 | 15:0 3OH * | 0.01 | 0.05 | 0.00 | 0.17 |
| 83 | 17:1 B | 0.01 | 0.05 | 0.00 | 0.18 |
| 86 | 17:0 CYCLO | 14.17 | 3.79 | 8.70 | 19.23 |
| 88 | 17:0 | 0.45 | 0.53 | 0.00 | 1.51 |
| 101 | 18:0 | 0.20 | 0.29 | 0.00 | 0.93 |
| 107 | 19:0 ISO * | 0.04 | 0.18 | 0.00 | 0.20 |
| 110 | 19:0 CYCLO C11-12 | 1.90 | 1.49 | 0.00 | 4.65 |
| 123 | SUMMED FEATURE 2 | 0.10 | 0.25 | 0.00 | 0.60 |
| 124 | SUMMED FEATURE 3 | 7.30 | 1.28 | 5.87 | 9.87 |
| 128 | SUMMED FEATURE 7 | 12.73 | 3.33 | 5.32 | 16.65 |

*Table 1. Comparison between two different preparative methods.*

| Organism | Our method | Hewlett-Packard method |
|---|---|---|
| | Similarity index | |
| *Enterobacter cloacae* | 0·807 | 0·547 |
| *Hafnia* | 0·760 | 0·513 |
| *Proteus mirabilis* | 0·316 | 0·159 |
| *Providentia stuartii* | 0·493 | 0·217 |
| *Pseudomonas aeruginosa* | 0·621 | 0·786 |
| *Salmonella choleraesuis* | 0·345 | 0·300 |
| *Staphylococcus aureus* | 0·120 | 0·190 |

The similarity indices of the same strains analysed both after additional cultivation on TSBA of the previously isolated colonies (Hewlett-Packard method) and directly from the routine isolation media (our method) were compared to quantify the influence of the growth media on the FAME pattern and consequently on the MIS identification.

were assumed to be whole numbers between 9000 and 20 000. The relationship between the ECL and the retention time of an unknown peak is expressed by the equation

$$\mathrm{ECL} = \frac{(\mathrm{Rt}_x - \mathrm{Rt}_n)}{(\mathrm{Rt}_{n+1} - \mathrm{Rt}_n)}$$

where $\mathrm{Rt}_x$ is the retention time of the FAME $x$, $\mathrm{Rt}_n$ is the retention time of the $\mathrm{C}(n:0)$, i.e. the straight-chain fatty acid that elutes before the FAME $x$ and $\mathrm{Rt}_{n+1}$ is the retention time of $\mathrm{C}(n+1):0$, i.e. the straight-chain fatty acid eluting just after the FAME $x$.

The identification of the fatty acid structure is performed using the ECL in a 'family plot' in which various fatty acid families are represented in relation to the straight-chain length ($x$ axes) and the retention time ($y$ axes). The ECL reported in the qualitative and quantitative standard mixture reports are those calculated under the optimum analytical conditions. The identification of the unknown peaks during the sample analysis is performed on the basis of these ideal parameters. In figure 3 the chromatogram, the analysis report and the comparison chart for the analysis of a pure strain of *Staphylococcus aureus* are shown. At the bottom of the report, in addition to the parameters reported for the calibration mixture, the genus and species and sub-species of the identified bacteria and the similarity index (SI) are given. The SI is a parameter that quantifies the reliability of MIS identification by measuring the overlapping of the FAME patterns of the sample and one of the various microorganisms stored in the computer library. The SI is a number which indicates how closely the FAME composition of an unknown sample compares with that of the library reference bacteria selected by the MIS. A value of SI = 1 means perfect overlapping and values less than 1 indicate that the patterns are not identical with a consequent higher inaccuracy of the result. The unknown strain is usually identified with the reference bacteria which gives the higher SI.

When the GC analysis is not good enough or the sample is too dilute, concentrated or contaminated, the identifica-

*Table 2. Day-to-day precision of the gas chromatic analysis.*

| Day | 1 | 2 | 3 | 4 | 5 | 6 | 7 | 8 | | |
|---|---|---|---|---|---|---|---|---|---|---|
| MIS identification | | | | Similarity index | | | | | M ± SD | CV% |
| *Pseudomonas aeruginosa* | 0·012 | 0·010 | 0·009 | 0·010 | 0·010 | 0·012 | 0·006 | 0·006 | 0·010 ± 0·001 | 10·0 |
| *Staphylococcus hominis* | 0·010 | 0·010 | 0·009 | 0·012 | 0·010 | 0·009 | 0·010 | 0·010 | 0·010 ± 0·0009 | 9·0 |
| *Kluyvera cryocrescens* | 0·446 | 0·335 | 0·402 | 0·387 | 0·381 | 0·401 | 0·392 | 0·386 | 0·391 ± 0·030 | 7·3 |
| *Proteus mirabilis* | 0·407 | 0·402 | 0·416 | 0·339 | 0·409 | 0·396 | 0·410 | 0·398 | 0·397 ± 0·022 | 5·7 |
| *Shigella dysenteriae* | 0·498 | 0·425 | 0·433 | 0·492 | 0·523 | 0·489 | 0·421 | 0·428 | 0·458 ± 0·037 | 8·0 |
| *Streptococcus epidermidis* | 0·156 | 0·182 | 0·203 | 0·187 | 0·198 | 0·189 | 0·204 | 0·210 | 0·191 ± 0·016 | 8·3 |
| *Klebsiella ozenae* | 0·483 | 0·492 | 0·482 | 0·473 | 0·480 | 0·462 | 0·478 | 0·475 | 0·478 ± 0·008 | 1·7 |

*Table 3. Bacterial identification obtained by reference method (microbiological identification) and gas chromatographic analyses.*

| Bacteriology laboratory identification | MIS identification | Tribe | Family |
|---|---|---|---|
| ***GRAM NEGATIVE AEROBES*** | | | |
| ***Acinetobacter calcoaceticus (n = 7)*** | ***Acinetobacter calcoac. (7)*** | | ***Neisseriaceae*** |
| ***Citrobacter freundii (n = 9)*** | ***Citrobacter freundii (5)*** | ***Salmonelleae*** | ***Enterobacteriaceae*** |
| | *Enterobacter cloacae (2)* | *Klebsielleae* | |
| | *Erwinia herbicola (1)* | *Erwinieae* | |
| | *Shigella dysenteriae (1)* | *Escherichieae* | |
| ***Escherichia coli (n = 63)*** | ***Escherichia coli (21)*** | ***Escherichieae*** | |
| | *Shigella dysenteriae (4)* | *Escherichieae* | |
| | *Kluivera cryocrescens (19)* | | |
| | *Citrobacter freundii (12)* | *Salmonelleae* | |
| | *Morganella morganii (5)* | *Proteeae* | |
| | *Enterobacter cloacae (2)* | *Klebsielleae* | |
| ***Enterobacter cloacae (n = 8)*** | ***Enterobacter cloacae (2)*** | ***Klebsielleae*** | |
| | *Serratia marcescens (1)* | *Klebsielleae* | |
| | *Escherichia coli (2)* | *Escherichieae* | |
| | *Kluyvera cryocrescens (1)* | | |
| | *Proteus mirabilis (1)* | *Proteeae* | |
| | *Morganella morganii (1)* | *Proteeae* | |
| ***Klebsiella ozaenae (n = 16)*** | ***Klebsiella ozaenae (6)*** | ***Klebsielleae*** | |
| | *Enterobacter cloacae (1)* | *Klebsielleae* | |
| | *Serratia marcescens (2)* | *Klebsielleae* | |
| | *Kluyvera cryocrescens (2)* | | |
| | *Shigella dysenteriae (2)* | *Escherichieae* | |
| | *Morganella morganii (1)* | *Proteeae* | |
| | *Salmonella choleraesuis (2)* | *Salmonelleae* | |
| ***Morganella morganii (n = 4)*** | ***Morganella morganii (1)*** | ***Proteeae*** | |
| | *Serratia marcescens (2)* | *Klebsielleae* | |
| | *Hafnia alvei (1)* | *Klebsielleae* | |
| ***Proteus mirabilis (n = 23)*** | ***Proteus mirabilis (20)*** | ***Proteeae*** | |
| | *Kluyvera cryocrescens (1)* | | |
| | *Enterobacter cloacae (2)* | *Klebsielleae* | |
| ***Providentia stuartii (n = 3)*** | ***Providentia stuartii (3)*** | ***Proteeae*** | |
| ***Salmonella choleraesuis (n = 7)*** | ***Salmonella choleraesuis (6)*** | ***Salmonelleae*** | |
| | *Erwinia herbicola (1)* | *Erwinieae* | |
| ***Pseudomonas aeruginosa (n = 45)*** | ***Pseudomonas aeruginosa (45)*** | | ***Pseudomonadaceae*** |
| ***Serratia liquefaciens (n = 1)*** | | ***Klebsielleae*** | ***Enterobacteriaceae*** |
| | *Acinetobacter calcoac. (1)* | | *Neisseriaceae* |
| ***GRAM POSITIVE AEROBES*** | | | |
| ***Staphylococcus aureus (n = 83)*** | ***Staphylococcus aureus (75)*** | | ***Micrococcaceae*** |
| | *Staphylococcus hominis (8)* | | |
| ***Staphylococcus hominis (n = 46)*** | ***Staphylococcus hominis (16)*** | | |
| | *Staphylococcus kloosii (15)* | | |
| | *Staphylococcus aureus (14)* | | |
| | *Acinetobacter calcoac. (1)* | | *Neisseriaceae* |
| ***Staphylococcus epidermidis (n = 9)*** | ***Staphylococcus epidermidis (5)*** | | |
| | *Staphylococcus aureus (2)* | | |
| | *Staphilococcus kloosii (2)* | | |
| ***Streptococcus pyogenes (n = 7)*** | ***Streptococcus pyogenes (7)*** | | ***Streptococcaceae*** |
| ***Streptococcus agalactiae (n = 9)*** | ***Streptococcus agalactiae (2)*** | | |
| | *Streptococcus pyogenes (4)* | | |
| | *Streptococcus faecalis (3)* | | |

*Table 3 – continued.*

| Bacteriology laboratory identification | MIS identification | Tribe | Family |
|---|---|---|---|
| ***Streptococcus equisimilis* (n = 2)** | *Streptococcus pyogenes (2)* | | |
| ***Streptococcus faecalis* (n = 10)** | ***Streptococcus faecalis (10)*** | | |
| ***Streptococcus β-hemoliticus* (n = 2)** | ***Streptococcus β-hemoliticus (2)*** | | |
| ***GRAM NEGATIVE ANAEROBES*** | | | |
| ***Bacteroides fragilis* (n = 7)** | ***Bacteroides fragilis (3)***<br>*Bacteroides oris (3)*<br>*Fusobacterium necrosis (1)* | | ***Bacteroidaceae*** |
| ***Fusobacterium necrosis* (n = 2)** | ***Fusobacterium necrosis (2)*** | | |
| ***GRAM POSITIVE ANAEROBES*** | | | |
| ***Clostridium difficile* (n = 1)** | ***Clostridium difficile (1)*** | | ***Bacillaceae*** |
| ***Streptococcus intermedius* (n = 2)** | *Streptococcus pyogenes (2)* | | ***Streptococcaceae*** |
| ***Propionibacterium acnes* (n = 1)** | ***Propionibacterium acnes (1)*** | | ***Propionibacteriaceae*** |

In the first column (on the left) the results of the microbiological identification (Genus and species) and the number of the samples tested are reported. In the second column the results of the MIS identification of the same strains are reported: results identical with the reference method are reported in the first line for each group in bold, while different results are listed below. The number of the respective cases is written in brackets. Under 'Tribe' and 'Family' both the cases of agreement (in bold) and disagreement between the two methods are indicated.

*Table 4. Distribution of the percentage of disagreement between microbiological and GC bacterial identification.*

| | Family | Genus | Species |
|---|---|---|---|
| **Gram negative aerobes** | | | |
| | 1/186 (0·5%) | 70/186 (37%) | 70/186 (37%) |
| **Gram positive aerobes** | | | |
| | 1/168 (0·6%) | 1/168 (0·6%) | 51/168 (30·3%) |
| **Gram positive anaerobes** | | | |
| | 0/4 | 0/4 | 2/4 (50%) |
| **Gram negative anaerobes** | | | |
| | 0/9 | 1/9 (11%) | 4/9 (44·5%) |

tion cannot be carried out and a comment is reported at the bottom of the sample report. A graphical representation of the library search by means of the comparison chart (figure 3) is printed if requested by the operator. The computer program can compare the unknown sample pattern with those of 1, 2, 3 or 4 bacteria stored in the library and print the relative comparison chart explaining the degree of similarity between the different bacteria. In the comparison chart all the fatty acids found in the unknown sample and the typical lipidic components of the reference bacteria stored in the library are listed. For each fatty acid listed, the percentage present in the unknown extract is compared with that of the same acid in the reference bacteria.

The distance between each FAME percentage found in the two compared bacteria is indicated visually in the comparison chart and an asterisk is printed when the two percentages are identical.

To verify the absence of interfering peaks, at the beginning of this study, reagents 1, 2, 3 and 4 used for the extraction and methylation were processed as microbial samples and injected into the gas chromatograph. None of them gave interfering peaks in the chromatogram.

In order to make the recommended standardized procedure easier, we analysed some strains by harvesting the pure colonies directly from MSA, MCK, MH, KV and PEA (the selective media commonly used in a bacteriology laboratory) and compared the results with those obtained after an additional passage of the previously isolated colonies on TSBA medium (as recommended by Hewlett-Packard).

As shown in Table 1, with the recommended procedure for some bacteria (*Proteus mirabilis, Providencia stuartii*) the SIs were lower than those found with our method. For the other strains the SIs were similar. In both cases the identification obtained with MIS agreed well with the reference method, so we decided to perform the extraction directly from the selective media with obvious advantages.

The day-to-day precision of the GC analysis was tested by injecting daily, for 8 days, the extracts of 7 different strains stored at +4 °C in tubes capped with a Teflon septum. The coefficient of variation (CV) of the SIs ranged from 1·7 to 10%. The results are shown in Table 2.

The results obtained from the GC analyses of 367 samples and the comparison with the reference method are summarized in Table 3.

According to Lennette *et al.* [8], the bacteria were classified into family, tribe, genus and species. Tribe is also considered for the Enterobacteriaceae family as a group including genus and species. All the bacteria tested were divided into four groups (aerobe Gram positive, aerobe Gram negative, anaerobe Gram positive and anaerobe Gram negative). As can be seen in Table 3, we obtained identical results in 65% of cases; all the microorganisms belonging to the families of Nesseriaceae, Pseudomonadaceae, Bacillaceae and Propionibacteriaceae were identified with the two methods. A lower percentage of agreement was obtained in the other families.

In Table 4 a detailed distribution of the percentage of disagreement in the bacterial identification is shown. The highest number of disagreements was found in the group of the aerobe Gram negative, especially within the Enterobacteriaceae family. Seventy cases (37%) of the total aerobe Gram negative tested (186) showed a different genus and species identification and consequently a different tribe attribution. Only one case was ascribed to a different family. In the aerobe Gram positive group, a very low percentage of disagreement was found for the family and genus classification (0·6%) whereas 30% of the identified samples disagreed for the species. For the anaerobe (Gram positive and Gram negative) the results are also reported even though the number of samples tested was low.

The greater disagreement in the identification of the Enterobacteriaceae family is probably due to the very similar lipidic compositions of the bacterial cell walls and to the similar metabolic behaviour of the bacteria included in this family. It must be emphasized that, for some bacteria, the MIS has a higher discriminatory power than the reference method; for example, the differentiation between *Kluyvera cryocrescens* and *Escherichia coli* is only possible with MIS, whereas with our reference method they are both classified as *Escherichia coli*.

### Discussion and Conclusion

The following points need some discussion: (1) the agreement between the MIS and the reference method; (2) the reproductibility of the MIS; (3) the need of an additional transplate of isolated colonies; and (4) some practical aspects concerning routine applications of MIS.

It is evident from our results that there is a certain degree of disagreement between the MIS and reference methods. This is variable within the different families studied. As expected, the highest degree of disagreement concerns the species identification, whereas it is lower for genus identification and almost absent for families identification. Even if chemical identification tests for bacteria are widely accepted, it is well known that most of them are based on very questionable chemical reactions (subjective detection, low sensitivity, etc.); in addition, the biological variability of bacteria accounts for the low reproducibility and accuracy. In some cases they have a discriminatory power much lower than MIS.

Hence the disagreement observed in our research is not surprising; more sensitive, reproducible and standardized methods for identification of bacteria are needed as standards or reference methods, with which new procedures should be compared. It must be stressed that the high discriminatory power of MIS, which is able to identify 150 different fatty acids, makes this system useful in taxonomic studies.

The following conclusions are suggested:

1. The reproducibility of the MIS is very good.
2. There is no need for an additional culture of isolated strains before injecting them into the gas chromatograph.
3. The system is easy to use because sample handling in the preparation of the extracts is minimized.
4. To obtain the final results and the identification of the bacterium, a shorter time is required when compared with traditional methods, which makes this system very suitable for emergencies. The total analysis time is about 2 h, including extraction, methylation and the gas chromatographic analysis, whereas with traditional tests 24–48 h are needed to confirm the identification of a pure strain.

5. The current methods require human interpretation, while the automated analysis improves the objectivity of the results and allows an easier comparison between different laboratories.

6. The autosampler allows the elimination of analyst errors, and thus reduces the waiting time between injections.

7. The cost of a gas chromatographic analysis (about $3 in Italy) is lower than that one for Enterotube II (Roche) or API (Ayerst) (about $5, $4 or $8·5) although the capital cost of the instrument is high.

8. The major practical disadvantage of this technique is its limited throughput (not more than 40 samples per 24 h), which prevents its use in a large routine laboratory, unless two or more instruments are installed.

Methodological and instrumental improvements to this technique will hopefully improve the quality of microbiological tests in routine laboratories and the classification of bacteria.

### Acknowledgement

This work was supported by Grant No. 561 of the Regione Lombardia: 'Precocious Diagnosis of Bacterial Infections By Gas Chromatographic and Mass Spectrometric Techniques: Development and Evaluation of Methodologies'.

### References

1. Kreig, N. R., in *Bergey's Manual of Systematic Bacteriology*, Kreig, N. R. (Ed.) (William and Wilkins, Baltimore, 1984), p. 1.
2. Moss, C. W., *Journal of Chromatography*, **203** (1981), 337.
3. Goodfellow, M. and Minnikin, D. E., in *Chemical Methods in Bacteriology Systematics*, Goodfellow, M. and Minnikin, D. E. (Eds) (Academic Press, London, 1985), p. 1.
4. Brondz, I. and Olsen, I., *Journal of Chromatography*, **379** (1986), 367.
5. Drucker, D. B., in *Microbiological Applications of Gas Chromatography*, Drucker, D. B. (Ed.) (Cambridge University Press, Cambridge, 1981), p. 251.
6. Asselineau, C. and Asselineau, J., in *Gas Chromatography–Mass Spectrometry. Applications in Microbiology*, Odham, G., Larsson, L. and Mardh, P. A. (Eds) (Plenum Press, New York, London, 1984), p. 57.
7. Bauer, A. W., Perry, D. M. and Kirby, W. M., *Archives of Internal Medicine*, **104** (1959), 208.
8. Lennette, E. H., Ballows, A., Hausler, W. J., Jr., and Truant, H. P., in *Manual of Clinical Microbiology* (American Society for Microbiology, Washington, DC, 1980).

